# Acceptability and Utility of a Smartphone App to Support Adolescent Mental Health (BeMe): Program Evaluation Study

**DOI:** 10.2196/47183

**Published:** 2023-08-28

**Authors:** Judith J Prochaska, Yixin Wang, Molly A Bowdring, Amy Chieng, Neha P Chaudhary, Danielle E Ramo

**Affiliations:** 1 Stanford Prevention Research Center Department of Medicine Stanford University Palo Alto, CA United States; 2 BeMe Health Miami, FL United States

**Keywords:** adolescents, mobile app, depression, anxiety, resilience, digital intervention, digital mental health, mobile phone

## Abstract

**Background:**

Adolescents face unprecedented mental health challenges, and technology has the opportunity to facilitate access and support digitally connected generations. The combination of digital tools and live human connection may hold particular promise for resonating with and flexibly supporting young people’s mental health.

**Objective:**

This study aimed to describe the BeMe app-based platform to support adolescents’ mental health and well-being and to examine app engagement, usability, and satisfaction.

**Methods:**

Adolescents in the United States, aged 13 to 20 years, were recruited via the web and enrolled between September 1 and October 31, 2022. App engagement, feature use, clinical functioning, and satisfaction with BeMe were examined for 30 days. BeMe provides content based on cognitive behavioral therapy, dialectical behavior therapy, motivational interviewing, and positive psychology; interactive activities; live text-based coaching; links to clinical services; and crisis support tools (digital and live).

**Results:**

The average age of the sample (N=13,421) was 15.04 (SD 1.7) years, and 56.72% (7612/13,421) identified with she/her pronouns. For the subsample that completed the in-app assessments, the mean scores indicated concern for depression (8-item Patient Health Questionnaire mean 15.68/20, SD 5.9; n=239), anxiety (7-item Generalized Anxiety Disorder Questionnaire mean 13.37/17, SD 5.0; n=791), and poor well-being (World Health Organization–Five Well-being Index mean 30.15/100, SD 16.1; n=1923). Overall, the adolescents engaged with BeMe for an average of 2.38 (SD 2.7) days in 7.94 (SD 24.1) sessions and completed 11.26 (SD 19.8) activities. Most adolescents engaged with BeMe’s content (12,270/13,421, 91.42%), mood ratings (13,094/13,421, 97.56%), and interactive skills (10,098/13,421, 75.24%), and almost one-fifth of the adolescents engaged with coaching (2539/13,421, 18.92%), clinical resources (2411/13,421, 17.96%), and crisis support resources (2499/13,421, 18.62%). Overall app engagement (total activities) was highest among female and gender-neutral adolescents compared with male adolescents (all *P*<.001) and was highest among younger adolescents (aged 13-14 years) compared with all other ages (all *P*<.001). Satisfaction ratings were generally high for content (eg, 158/176, 89.8% rated as helpful and 1044/1139, 91.66% improved coping self-efficacy), activities (5362/8468, 63.32% helpful and 4408/6072, 72.6% useful in coping with big feelings), and coaching (747/894, 83.6% helpful and 747/894, 83.6% improved coping self-efficacy). Engagement (total activities completed) predicted the likelihood of app satisfaction (*P*<.001).

**Conclusions:**

Many adolescents downloaded the BeMe app and completed multiple sessions and activities. Engagement with BeMe was higher among female and younger adolescents. Ratings of BeMe’s content, activities, and coaching were very positive for cognitive precursors aimed at reducing depression and anxiety and improving well-being. The findings will inform future app development to promote more sustained engagement, and future evaluations will assess the effects of BeMe on changes in mental health outcomes.

## Introduction

### Background

The state of adolescent mental health has been steadily declining, and the COVID-19 pandemic ushered in a new set of challenges. Across the 10 years preceding the pandemic, feelings of persistent sadness and hopelessness as well as suicidal thoughts and behaviors increased by approximately 40% among young people, according to the Centers for Disease Control and Prevention’s Youth Risk Behavior Surveillance System [1,2]. In 2021, about 4 in 10 American high school students reported feeling persistently sad or hopeless over the last year, and 1 in 11 attempted suicide [3]. Since 2019, emergency department visits for mental health conditions among adolescents aged 13 to 17 years have increased annually [4]. A 2021 report from the Surgeon General called adolescent mental health a national crisis and made a series of recommendations to support youth, including empowering youth and their families to recognize, manage, and learn from difficult emotions; ensuring that every child has access to high-quality, affordable, and culturally competent mental health care; and promoting equitable access to technology that supports the well-being of children and youth [5].

Adolescence is the developmental period that begins with the onset of puberty and is defined by the American Academy of Pediatrics as spanning ages 11 to 21 years; it is a phase during which a young individual transitions from childhood to adulthood [6]. Throughout this developmental period, adolescents experience unique stressors, including social transitions, physical and emotional changes, and other potentially overwhelming life challenges, which may benefit from adolescent-targeted psychological guidance and support. Although there are a multitude of psychotherapeutic approaches that have a strong evidence base for enhancing adolescent mental health [7-9], many young people experience barriers in accessing traditional in-person therapy (eg, stigma, finances, transportation barriers, and inconvenient appointment times) [10-12], and half of the children diagnosed with a mental health condition in the United States do not receive treatment [13]. Therefore, alternative methods for care service delivery are required.

Digital health tools have a significant potential to increase adolescents’ access to mental health support. Although early studies on web-based mental health services failed to demonstrate effectiveness in increasing help-seeking behavior among adolescents [14], the surge in mobile app development over the past decade has changed the landscape of digital mental health. Recent estimates suggest that there are >40,000 health and medical apps on leading app sites [15]. With 95% saturation of smartphone use among adolescents [16], mobile apps in particular may have the ability to provide desirable, accessible, and affordable support to adolescents when and where they are most likely to consume this support.

A recent systematic review indicated that most existing digital interventions (mobile and otherwise) are not evidence based, and there is inconclusive support for their effectiveness on mental health concerns other than anxiety and depression [17]. However, a separate, recent review and meta-analysis of 80 studies describing 83 mobile health interventions based on evidence-based principles found symptom improvement for a variety of psychological disorders, improved general well-being, and reduced distress [18]. Indeed, evidence-based digital services may offer the best opportunity to enhance outcomes among adolescents. For example, brief coping skills delivered digitally have been shown to improve mood and support young people in managing difficult moods without finding them unbearable, thereby preventing mental health challenges [19]. In addition, positive psychology skills focused on enhancing positive emotions and reducing negative emotions, when delivered via a smartphone app, can buffer older adolescents against loneliness and depression, improve sleep quality, and aid adjustment to college [20].

Mixed findings in the literature for adolescent mental health digital interventions may also be partly because of the combined assessment of bot coaching with human support [18]. The combination of digital tools and live human connection may hold particular promise for resonating with and flexibly supporting adolescent mental health. The addition of synchronous human support through skills-based coaching appears to improve adherence to and outcomes from digital mental health interventions as well as lower dropout among adolescents [17]. For example, live counselor contact was associated with improved clinical outcomes from a smoking cessation intervention delivered via Facebook to young adults [21]. Peer-to-peer counseling from paraprofessionals in a digital environment aided the delivery of evidence-based skills to undergraduate college students [22]. Tools that combine digital coping–skills delivery with live human connection for extra support and crisis management have the potential to help adolescents across a range of mental health challenges.

Despite the profound potential of digital health tools to address gaps in adolescent mental health care, initiation and continued app engagement remains suboptimal. Although some digital mental health tools have reported positive user engagement and adherence under certain conditions [17,23], many apps fail to retain users beyond initial registration [24], with evidence of poor user experience and negative perceptions of app usefulness among adolescents [25,26]. In recent years, there has been increased advocacy for the critical importance of involving people with lived experience in treatment development and, specifically, involving young people in the development of digital health products for adolescents [27,28]. Focus groups with adolescents have identified interest in strengths-based mobile health coaching and structured, supported web-based peer-to-peer interactions [29]. For digital mental health tools, adolescents have also identified interest in self-directed learning, multimedia (eg, audio and video components), and content diversity as opposed to focusing on a singular health issue [30]. Designing the look and functionality of mobile mental health tools according to adolescent preferences is likely to enhance reach and engagement.

With a purposeful focus on adolescent experience in its app design, BeMe Health, a digital mental health company, engages a Teen Advisory Board in partnership with adolescent clinical scientists and technology product and safety experts. The BeMe app was designed to support adolescent mental health by combining digital support through content and interactive care activities, live human connection through skills-based coaching with paraprofessionals, early identification and facilitation of clinical services as needed, and in-app digital crisis support tools linked to live crisis support as needed. Skills and coaching support in the BeMe app are based on interventions that have a strong and sound evidence base with adolescents, including cognitive behavioral therapy [31], dialectical behavior therapy [32], acceptance and commitment therapy [33], mindfulness-based self-compassion [34], positive psychology [35], and motivational interviewing [36].

### Objectives

The goal of this first large-scale evaluation of the BeMe app was to assess acceptability and utility among adolescents throughout the United States. We examined adolescent use patterns over 30 days on the BeMe platform, satisfaction and perceived helpfulness across BeMe’s features, and predictors of app engagement.

## Methods

### Participant Recruitment

Deidentified data were obtained within the BeMe app from adolescents aged 13 to 20 years between September 1 and October 31, 2022, when the beta version of the app included all basic features to characterize and support adolescent mental health. Participant use of the BeMe app was tracked for 30 days from the day of enrollment. Participants learned about BeMe from various channels, including unpaid posts on social media platforms, social media or other web-based platform advertisements (maximum daily budget was US $1000), or from organic channels such as word of mouth from other adolescents. Social media posts included adolescent-centric images and phrases that shared BeMe’s focus on adolescent mental health and highlighted specific features (eg, coaching). Posts were linked to BeMe’s website (BeMe [37]) that shared links to download the app in the iOS or Android app stores.

Upon downloading the app, adolescents registered on the app and were asked to read and accept BeMe’s Terms of Service and Privacy Policy, written in adolescent-facing language and at a 5th-grade reading level. The terms of service (Multimedia Appendix 1) indicated that the data would be used to improve BeMe’s services. The adolescents then completed a profile and were instructed to use the app freely. Assessments were embedded throughout the app experience. To avoid coercion that would affect app engagement and because all metrics used in this study were embedded in the intervention rather than requiring separate time to be spent on completion, adolescents were not given any compensation for using BeMe. Prior research with adolescents and parents has questioned whether the use of incentives for adolescents might lead to invalid results through misrepresentation [38-40].

### Ethical Considerations

The project was deemed to be a program evaluation exempt from review by Stanford’s Institutional Review Board. Program evaluations follow a systematic method for collecting and analyzing information with the intent of answering questions about the effectiveness and efficiency of a specific program, in this case a digital health program.

### Intervention

#### Overview

The BeMe platform was designed to improve well-being and address the mental health needs of all teens across the specific need and acuity spectra. BeMe was designed to provide both preventive skills that promote resilience and thriving among all teens as well as provide interventions for common clinical symptoms such as depression and anxiety; support healthy habit formation and behavior change (eg, improve sleep and reduce or quit substance use); and support linkage to clinical services and crisis support for those teens who need it. BeMe accepts any teen on its platform (there are no exclusion criteria for enrollment other than age), and it functions as a primary, secondary, and tertiary prevention program depending on adolescents’ needs at enrollment.

BeMe was designed by a combination of adolescent advisors and experts in the fields of behavioral science, adolescent clinical intervention, medicine, crisis support, mobile apps, and child- and adolescent-focused technology products. During the design phase, BeMe’s Teen Advisory Board had 68 members across the United States, all of whom had lived experience with the topics addressed in the app and some with clinical symptoms of anxiety, depression, or other common mental health concerns. The adolescents informed the look, tone, and design of BeMe’s overall app as well as its specific features. BeMe’s app was open to users aged ≥13 years in accordance with data privacy and protection regulations. Caregiver consent was not required for enrollment in the BeMe app; however, it was required for an adolescent to engage in clinical services through the BeMe app. The beta version of the BeMe app contained the following 5 main features (refer to Multimedia Appendix 2 for samples): content, activities, coaching, clinical services, and crisis support.

#### Content

*Content* emphasized coping and resilience-building skills based on evidence-based strategies, including cognitive behavioral therapy [31], dialectical behavior therapy [32], acceptance and commitment therapy [33], mindfulness-based self-compassion [34], positive psychology [35], and motivational interviewing [36]. Content included resilience and coping skills for all adolescents, skills for coping with specific mental health conditions (eg, depression and anxiety), and skills specific to adolescent-centric identities and challenges (eg, dealing with a breakup, challenges with friends or caregivers, and school stress). Content was multimodal, including text screens, videos with and without music or voice-overs, carousels with text or images, and images with dynamic features. Content was tagged based on the primary focus of each piece (ie, validation, general psychoeducation, skill building, and inspiring joy), the evidence-based strategy it drew upon, and the theme (eg, friend fights) and was assigned a learning objective (eg, to improve communication). The tagging system enabled the labeling of specific content pieces from the overall content library so that adolescents could navigate to different types of content based on preferences and needs.

#### Activities

*Activities* provided interactive tools for practicing resilience and coping skills. Designed collaboratively by BeMe and its Teen Advisory Board, activities included: (1) mood ratings, (2) interactive skills, and a (3) a community-based skill. Mood ratings included both *simple* and more complex *interactive* versions. Simple mood ratings included a single item with 4 responses ranging from positive (“I’m great”) to negative (“I’m really struggling”). Interactive ratings used the phone’s camera and digital stickers to encourage teens to take a selfie and label their mood and shared these data back with adolescents in a separate journal section of the app. Interactive skills based on cognitive and behavioral coping skills, mindfulness practice, and positive psychology skills, including those described by Bruehlman-Senecal et al [20] and others. A community-based skill was designed to practice sending and receiving “good vibes” with other adolescents (to promote a sense of community without actual social features).

#### Coaching

*Coaching* was delivered by BeMe coaches live in the app via text messaging. The coaches were graduate- and undergraduate-level paraprofessionals trained in a program developed by BeMe’s clinical leadership team. Training included a combination of didactic training, role playing, and supervisor observation before working on the platform as well as ongoing weekly individual and group supervision and live supervision during all shifts. Adolescents could message at any time of the day or night with a preset message or an open-ended message of their choice. During the study period, BeMe coaches responded to adolescents for 14 hours per day. All adolescents could message an unlimited number of times on any topic of their choice during the study period. After a coaching session, adolescents received a feedback form with session ratings. Coaches tagged conversations with any number of topics designed by BeMe’s clinical leadership, or as “other.”

#### Clinical Services

Adolescents could connect to a licensed therapist through the BeMe app. Successful linkage to treatment required registration, verified parental consent, and scheduling via telephone or text messages.

#### Crisis Support

Adolescents could self-navigate or be directed by a coach to crisis support services, including 3 live options: a crisis hotline staffed 24/7, the Crisis Text Line, or the Trevor Project Crisis Support services. Adolescents could also complete a digital safety plan based on evidence-based suicide prevention support tools for adolescents [41].

The beta version was not designed to be used in any specific pattern or length. Adolescents were given free access to the app and could navigate through any or all features as they chose.

### Measures

#### Sample Characteristics

Upon enrollment in the BeMe app, adolescents reported their age, preferred pronouns, interests from a list codeveloped with BeMe’s Teen Advisory Board, and “onboarding topics” adolescents indicated that they would like to explore on the BeMe app. Adolescents could select any number of interests and topics.

#### Clinical Functioning

At any point in their BeMe journey, adolescents could opt to complete assessments of their clinical functioning regarding anxiety, depression, stress, and overall well-being. The tools were selected based on their evidence of use with adolescents. There was no particular time in the engagement process in which the assessments were administered, and participants self-selected to complete any, all, or none of the assessments and could repeat the assessments. The first instance of a completed assessment was analyzed in this study to characterize adolescent functioning. With reference to the past 2 weeks, adolescents completed the 7-item Generalized Anxiety Disorder Questionnaire [42] with scores ranging from 0 to 21; scores from 5 to 9 indicated mild anxiety, scores from 10 to 14 indicated moderate anxiety, scores from 15 to 19 indicated moderately severe anxiety, scores >15 indicated moderately severe anxiety, and scores >20 indicated severe anxiety. Adolescents could also complete the 8-item Patient Health Questionnaire (PHQ-8) adolescent version [43]. Again, with reference to the past 2 weeks, PHQ-8 total scores ranged from 0 to 24; scores from 5 to 9 indicated mild depression, 10 to 14 indicated moderate depression, 15 to 19 indicated moderately severe depression, and >19 indicated severe depression. The 4-item Perceived Stress Scale [44] assessed participants’ self-reported stress level over the past month on a scale ranging from 0 (“never”) to 4 (“very often”; total score range 0-16). The World Health Organization–Five Well-being Index (WHO-5), widely used with adolescents [45], was used to assess overall well-being during the past 2 weeks. The WHO-5 scores range from 0 to 25 and are multiplied by 4 to yield a well-being score between 0 and 100. Scores ≤50 indicate poor well-being, and scores <28 indicate depression, including in adolescent samples [46].

#### Engagement

Engagement was measured at the individual user level and the app feature level. Heterogeneity in engagement metrics has been reported in previous studies on mental health apps [47]. In this study, we used standard engagement metrics to report on at least minimal use (ie, days with any engagement on the app), number of times on the app (ie, number of unique sessions), and number of unique features used (ie, activities) [48]. Engagement was also calculated at the app feature level as the number of times each feature was used in a month among those who engaged in that feature. Content engagement was defined as the completion of the entirety of a piece of content (eg, scrolling through all screens of a carousel or watching an entire video). Engaged content was summarized by an intervention skillset coded as acceptance and commitment therapy, behavioral activation, cognitive and behavioral therapy, dialectical behavioral therapy, emotion regulation and distress tolerance skills, interpersonal effectiveness skills, mindfulness practice, motivational interviewing techniques, positive psychology skills, and trauma-informed care skills. Activity engagement was summarized for drawing practice, mindfulness skills, distress tolerance or emotion regulation skills with the phone’s camera, or movement-based skills with the phone’s camera. Interactive mood rating feature moods were tallied for each of the 21 moods across all activities completed. The mood list was developed by the study’s last authors (NC and DR) in collaboration with BeMe’s Teen Advisory Board. The coaching topics were coded by the BeMe coaches at the end of each session. Topic data were only available for approximately half of the study period (October 7, 2022, to November 30, 2022), so the sample of coaching sessions available for analysis was not the full sample of completed sessions during this time. The number of times clinical supports and safety plan resources (digital safety plan and crisis support services) were accessed was tallied. For the adolescents who completed a digital safety plan, we computed the average number of endorsed reasons for living, crisis warning signs, ways to make their environment safe, and coping skills.

#### Satisfaction and Helpfulness

Satisfaction and helpfulness were measured at the feature level. After select pieces of content created by BeMe, adolescents were asked to rate the content on perceived helpfulness and confidence that they would use the skill outside of the BeMe app (self-efficacy). After select pieces of content, adolescents rated whether they felt hopeful (hope), learned something about themselves (self-identity), the content was helpful to their self-esteem (self-esteem), felt less alone (social connection), or felt relaxed and grounded (relaxation; all yes or no). Activities were rated similarly based on perceived helpfulness and utility in coping with a big feeling. The percentage of polls with yes responses was tallied for each response type. Coaching sessions were rated on a 6-point scale (0-5 stars), perceived helpfulness, and self-efficacy (yes or no).

### Analyses

#### Sample Characteristics and Use Patterns

Descriptive statistics were used to characterize adolescent demographics for the full sample, clinical functioning among the subset that self-selected to complete the assessments, and overall app engagement patterns.

#### Relationship Between Individual Characteristics and Engagement

Logistic regression was used to evaluate predictors of overall app engagement (ie, total activities accessed). The dependent variable was the number of activities completed in 30 days, coded as high or low based on the sample median (7 activities). The independent variables (IVs) were pronouns, age, and overall well-being (WHO-5 total score). *P* values and CIs determine whether the association between overall app engagement and each term in the model is statistically significant. All IVs were included in the model at once because we lacked an underlying theory to guide model selection. This approach was adopted to prevent bias toward small *P* values and large parameter estimates [49].

#### Satisfaction With BeMe’s Content and Features

Engagement with content and activities was evaluated at the feature level, and the proportion of “yes” responses was computed for each content or activity type. Coaching satisfaction was evaluated among the proportion of adolescents who engaged with coaching at least once. The patterns of engagement, topics, and ratings were computed for this subsample.

#### Relationship Between Individual Characteristics and Satisfaction

A logistic regression model was used to evaluate the predictors of satisfaction and helpfulness. The dependent variable was the presence of at least 1 “yes” response on a postactivity survey (eg, after a piece of content or activity). IVs were pronouns, age, the WHO-5 total score, and total activities completed dichotomized, consistent with the strategy described in Relationship Between Individual Characteristics and Engagement. *P* values and CIs determine whether the association between satisfaction and helpfulness and each term in the model was statistically significant.

#### Power Estimation

Planned analyses included at least 2 regression models (1 predicting engagement and 1 predicting satisfaction), with up to 6 IVs, including age, preferred pronouns, depression, anxiety, overall well-being, and perceived stress. A multivariate regression analysis with 6 IVs testing a partial *R*^2^ of 0.1 in each IV with an error probability of 0.05 and 95% power requires a sample size of at least 195. A sample size of up to 2000 was deemed sufficient to detect a small effect size for each IV in the 2 models. Relatively low sample sizes in depression, anxiety, and well-being measures compared with other IVs yielded their exclusion from the final models; thus, we expect the final sample size of >13,000 to be adequately powered to report both the final regression models and a series of frequency and proportion results shared in this study.

## Results

### Sample Characteristics and Use Patterns

In the 2-month enrollment period, 13,421 adolescents were enrolled in the BeMe app. The characteristics of the sample are presented in Table 1.

**Table 1:** Sample characteristics (N=13,421).

Characteristics			Values
**Age (years)**			
	Mean (SD)	15.04 (1.7)	
	Median (IQR)	15 (14-16)	
	13-14, n (%)	5977 (44.53)	
	15-16, n (%)	4537 (33.81)	
	17-18, n (%)	2425 (18.07)	
	19-20, n (%)	482 (3.59)	
**Pronouns, n (%)**			
	She/her	7612 (56.72)	
	He/him	1468 (10.94)	
	They/them	1391 (10.36)	
	Other/no response	2950 (21.98)	
**Interests, n (%)**			
	Music	10,966 (81.71)	
	Art	7821 (58.27)	
	Food	7231 (53.88)	
	Animals	7102 (52.92)	
	Beauty	6721 (50.08)	
	Fashion	6193 (46.14)	
	Reading	5941 (44.27)	
	Nature	5750 (42.84)	
	Photography	5395 (40.20)	
	Writing	5383 (40.11)	
	Gaming	5362 (39.95)	
	LGBTQIA^a^	5331 (39.72)	
	Travel	4874 (36.32)	
	Dance	4408 (32.84)	
	Anime	4157 (30.97)	
	Sports	3858 (28.75)	
	Science	2547 (18.98)	
	Entrepreneurship	1327 (9.89)	
	Climate	1131 (8.43)	
	Auto	768 (5.72)	
**Goals for using BeMe, n (%)**			
	Boosting happiness	10,049 (74.88)	
	Building relationships	9000 (67.06)	
	Dealing with stressors	9337 (69.57)	
	Discovering identity	6757 (50.35)	
	Finding ways to cope	8025 (59.79)	
	Living mindfully	5706 (42.52)	
	Managing mood	9314 (69.40)	
	Navigating life transition	4923 (36.68)	
**Depression symptoms (PHQ^b^ score; n=238)**			
	Mean (SD)	15.69 (5.91)	
	Median (IQR)	17 (13-20)	
	No depression (scores: 0-4), n (%)	18 (7.56)	
	Mild (score: 5-9), n (%)	17 (7.14)	
	Moderate (score: 10-14), n (%)	51 (21.43)	
	Moderately severe (score: 15-19), n (%)	85 (35.71)	
	Severe (score: 20+), n (%)	67 (28.15)	
**Anxiety symptoms (GAD^c^ score; n=791)**			
	Mean (SD)	13.37 (5.01)	
	Median (IQR)	14 (10-17)	
	None to normal (score: 0-4), n (%)	52 (6.57)	
	Mild (score: 5-9), n (%)	125 (15.80)	
	Moderate (score: 10-14), n (%)	259 (32.74)	
	Severe (score: 15-21), n (%)	355 (44.88)	
**Overall well-being (WHO-5^d^ score; n=1322)**			
	Mean (SD)	30.15 (16.06)	
	Median (IQR)	28 (20-40)	
	≤50, n (%)	1172 (88.65)	
	≤28, n (%)	747 (56.51)	
**Perceived stress (PSS^e^ score; n=638)**			
	Mean (SD)	10.62 (2.56)	
	Median (IQR)	11 (9-12)	
	≥6, n (%)	623 (97.65)	

^a^LGBTQIA: lesbian, gay, bisexual, transgender, queer, intersex, asexual, and similar minority.

^b^PHQ: Patient Health Questionnaire.

^c^GAD: Generalized Anxiety Disorder Questionnaire.

^d^WHO-5: WHO-5 Well-being Index.

^e^PSS: Perceived Stress Scale.

The average age of the adolescents was 15.04 (SD 1.7) years, and they more often identified with female pronouns (7612/13,421, 56.72%) than male pronouns (1468/13,421, 10.94%), gender-neutral pronouns (1391/13,421, 10.36%), or other or decline to answer (2950/13,421, 21.98%). Onboarding topics selected by a majority of adolescents were boosting happiness (10,049/13,421, 74.88%), dealing with stressors (9337/13,421, 69.57%), managing mood (9314/13,421, 69.40%), building relationships (9000/13,421, 67.06%), and generally finding ways to cope (8025/13,421, 59.79%); 50.35% (6757/13,421) of the adolescents selected discovering your identity, and although less common, over a third selected living mindfully (5706/13,421, 42.52%) and navigating a life transition (4923/13,421, 36.68%). The most common interests endorsed by the adolescents were music (10,966/13,421, 81.71%), art (7821/13,421, 58.27%), food (7231/13,421, 53.88%), and animals (7102/13,421, 52.92%).

### Engagement

Table 2 displays overall and feature-level engagement in the BeMe app over 30 days. On average, the adolescents engaged with BeMe for >2 days (mean 2.38, SD 2.72; median 1, IQR 1-3; range 1-30), in 8 sessions (mean 7.94, SD 24.14; median 3, IQR 2-7; range 1-1750), and completed >11 activities (mean 11.26, SD 19.81; median 7, IQR 4-12; range 1-776). Adolescents were most likely to engage in simple mood ratings (13,094/13,421, 97.56%), content (12,270/13,421, 91.42%), and interactive skills (10,098/13,421, 75.24%). Almost one-fifth of the adolescents completed a coaching chat session (2539/13,421, 18.92%), explored clinical resources (2499/13,421, 18.62%), and engaged in safety planning (1129/13,421, 8.41%).

**Table 2:** Overall and specific feature engagement (N=13,421).

Variable				Values, mean (SD)		Values, median (IQR)		Values, n (%)
**Overall app engagement^a^**								
	**Days engaged (range** **1-30)**			2.38 (2.72)		1 (1-3)		13,421 (100)
		1	1 (0)		1 (1-1)		6962 (51.87)	
		2-3	2.35 (0.48)		2 (2-3)		4233 (31.54)	
		≥4	6.77 (4.35)		5 (4-8)		2226 (16.59)	
	App sessions (range 1-1750)			7.94 (24.14)		3 (2-7)		13,421 (100)
	Activities (range 1-776)			11.26 (19.81)		7 (4-12)		13,421 (100)
**Specific feature engagement^a^**								
	Content views (range 1-505)			4.59 (11.36)		2 (1-5)		12,270 (91.42)
	Simple mood rating (range 1-128)			2.61 (3.49)		2 (1-3)		13,094 (97.56)
	Interactive skills (range 1-75)			2.58 (3.09)		2 (1-3)		10,098 (75.24)
	Interactive mood rating (range 1-80)			1.82 (2.57)		1 (1-2)		6677 (49.75)
	Community skills (range 1-73)			1.75 (2.72)		1 (1-2)		1616 (12.04)
	Coach session (range 1-20)			1.47 (1.27)		1 (1-1)		2539 (18.92)
	Clinical service resource clicks (range 1-19)			1.40 (0.97)		1 (1-1)		2411 (17.96)
	**Crisis resource views (range 1-25)**			1.52 (1.25)		1 (1-2)		2499 (18.62)
		Safety plan completed (yes or no)	N/A^b^		N/A		1129 (8.41)	

^a^Teens were not given guidance as to how to navigate through BeMe’s features, and there were no limits on the number of activities and interactions with each feature (eg, content and mood rating) possible during the 30-day study period.

^b^N/A: not applicable.

Overall, 91.53% (12,285/13,421) of adolescents accessed 66,345 pieces of content (content views: mean 4.59, SD 11.35, median 2, IQR 1-5; range 1-505). Adolescents engaged most with content grounded in dialectical behavior therapy emotion regulation and distress tolerance skills (16,920/66,345, 25.5% of all content viewed), positive psychology skills (14,346/66,345, 21.62% of all content viewed), cognitive and behavioral therapy coping skills (16,920/66,345, 25.5%), and mindfulness-based self-compassion skills (8650/66,345, 13.04%). Lower engagement was found with content grounded in acceptance and commitment therapy skills (5669/66,345, 8.54%), dialectical behavioral therapy interpersonal effectiveness and social skills (5810/66,345, 8.76%), motivational interviewing skills (1693/66,345, 2.55%), behavioral activation (1091/66,345, 1.64%), and skills grounded in trauma-informed care (774/66,345, 1.17%).

Among those adolescents who engaged in each type of interactive activity, engagements were, on average, as follows: 2.61 (SD 3.49; median 2, IQR 1-3) simple mood ratings, 2.58 (SD 3.09; median 2, IQR 1-3) interactive skills, 1.82 (SD 2.57; median 1, IQR 1-2) interactive mood ratings, and 1.75 (SD 2.72; median 1, IQR 1-2) community-based skills. Considering specifically interactive skills, adolescents primarily engaged with those that used the phone’s camera to complete a distress tolerance skill (24,944/44,698, 55.81%) or a pleasurable activity (11,963/44,698, 26.76%). Considering interactive mood ratings (n=19,800 rating completed), intraclass correlation in a 2-way mixed effects model using a consistency definition showed a significant correlation among mood ratings across individuals (intraclass correlation 0.427, CI 0.42-0.44; *P*<.001), so frequency was tallied for all mood ratings. The most frequently identified moods were low arousal moods, including lonely (2315/19,800, 11.69%), low (2280/19,800, 11.52%), relaxed (2160/19,800, 10.91%), hurt (2074/19,800, 10.47%), bored (1947/19,800, 9.83%), and depressed (1755/19,800, 8.86%). The least endorsed moods were those with higher arousal, including pumped (368/19,800, 1.86%), furious (390/19,800, 1.97%), excited (413/19,800, 2.09%), and energetic (447/19,800, 2.26%). Other endorsed moods included chill (1681/19,800, 8.49%), insecure (1460/19,800, 7.37%), anxious (1223/19,800, 6.18%), grateful (1129/19,800, 5.7%), happy (1147/19,800, 5.79%), content (1086/19,800, 5.48%), motivated (1031/19,800, 5.21%), cheerful (957/19,800, 4.83%), rejected (943/19,800, 4.76%), frustrated (805/19,800, 4.07%), and pissed (521/19,800, 2.63%).

Among the adolescents who engaged with coaching, they averaged 1.47 (SD 1.27; median 1, IQR 1-1) sessions. The topics were coded for 1817 coaching sessions during the study period. Anxiety was the most common topic raised in coaching (649/1817, 35.72%) and was more than twice as frequent as the next most popular topic. The relationship concerns of different types were also common: romantic (304/1817, 16.73%), friend (264/1817, 14.53%), and family (187/1817, 10.29%). School-related topics (eg, workload and managing school stress) were raised in 14.42% (262/1817) of the coaching conversations. Adolescents also sought coaching on self-esteem (160/1817, 8.81%); depression and sadness (153/1817, 8.42%); body image (80/1817, 4.4%); issues related to lesbian, gay, bisexual, transgender, queer, intersex, asexual, and similar minority (LGBTQIA) identity (73/1817, 4.02%); anger (61/1817, 3.36%); suicidality (47/1817, 2.59%); bullying (46/1817, 2.53%); and self-harm (37/1817, 2.04%). The less common topics were related to learning new skills (35/1817, 1.93%), grief (29/1817, 1.60%), loneliness (19/1817, 1.05%), neurodiversity (15/1817, 0.83%), abuse or neglect (11/1817, 0.61%), harassment or assault (9/1817, 0.5%), racism or discrimination owing to ethnicity (2/1817, 0.11%), and substance use (2/1817, 0.11%).

The adolescents who sought out safety and clinical resources selected on average 1.52 (SD 1.25; median 1, IQR 12) safety resources (crisis supports and digital safety plan) and 1.35 (SD 0.80; median 1, IQR 1-1) clinical resources. Almost half (1129/2499, 45.18%) of those who engaged with the safety resources completed a digital safety plan, averaging 5.07 (SD 2.62) out of 12 reasons for living, 7.05 (SD 3.64) out of 17 crisis warning signs, 2.25 (SD 1.96) out of 8 ways to make their environments safe, and 3.69 (SD 2.28) out of 9 coping skills to use when in crisis.

### Predictors of BeMe App Engagement

**Figure 1:**
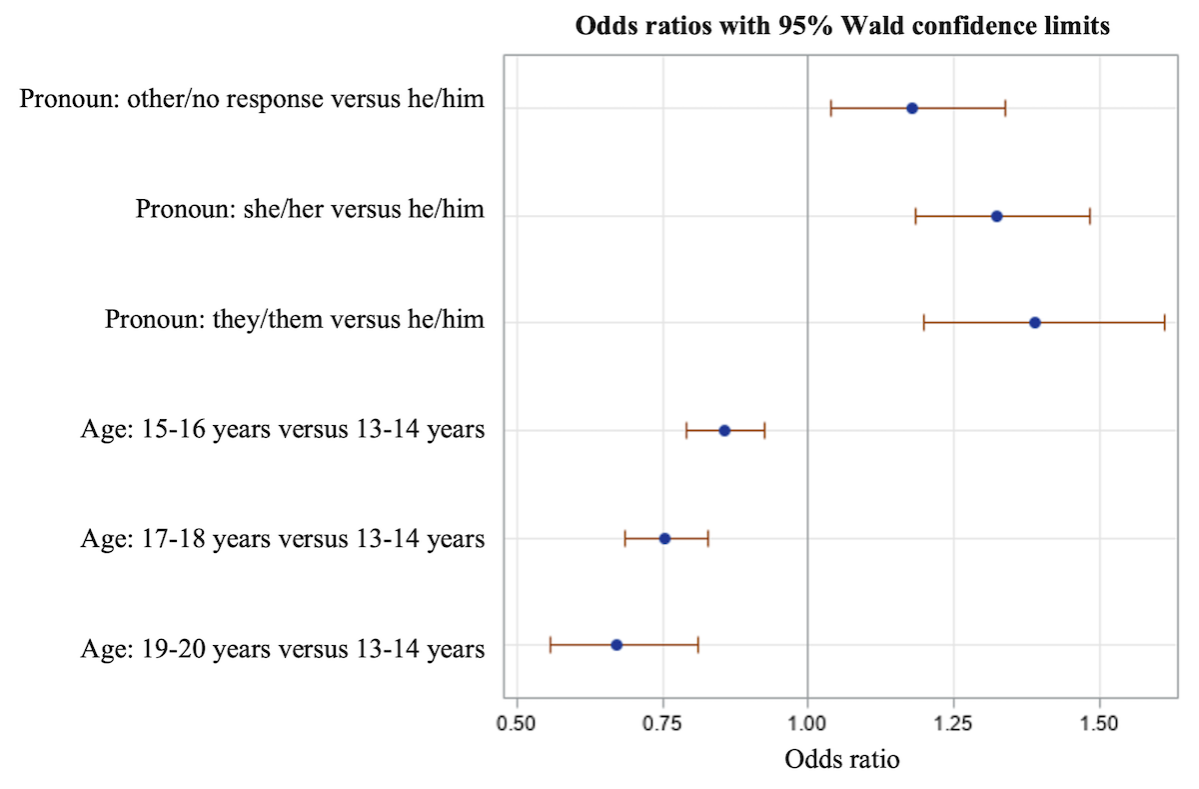
Odds ratios and CIs for a logistic regression model testing the effects of pronouns and age on app engagement (total activities over 30 days; high vs low engagement).

To examine the predictors of overall app engagement, with high versus low engagement (<7 vs >7 activities completed over 30 days) as the outcome, we first ran a logistic model including pronouns, age, and overall well-being (WHO-5 total score). The WHO-5 measure was not predictive in the model (*P*=.61) and had a lot of missing data (completed by 1322 adolescents); therefore, we reran the model with only pronouns and age as predictors (Figure 1; Table 3). Predictors were pronouns in 4 categories (she/her, he/him, they/them, and other/no response), with he/him as the reference group, and age in 4 categories, with 13-14 as the reference group (N=13,421). The findings indicated that compared with male-identified adolescents, female-identified adolescents were 32% more likely to be in the high engagement group (odds ratio [OR] 1.324, 95% CI 1.184-1.482; *P*<.001), gender-neutral adolescents were 39% more likely to be in the high engagement group (OR 1.39, 95% CI 1.199-1.61; *P*=.03), and those who did not indicate pronouns or chose other were 18% more likely to be in the high engagement group (OR 1.179, 95% CI 1.04-1.337; *P*=.01). In addition, adolescents in the youngest age group were more likely to be in the highest engagement group than the 3 older age groups (all *P*<.001).

**Table 3:** Logistic regression model predicting high versus low app engagement (total activities completed; N=13,421).

Effect			Odds ratio (95% CI)		*P* value
**Pronoun**					
	Other/no response vs he/him	1.179 (1.04-1.337)		.01	
	She/her vs he/him	1.324 (1.184-1.482)		<.001	
	They/them vs he/him	1.390 (1.199-1.61)		<.001	
**Age (years)**					
	15-16 vs 13-14	0.855 (0.792-0.924)		<.001	
	17-18 vs 13-14	0.754 (0.686-0.829)		<.001	
	19-20 vs 13-14	0.673 (0.558-0.811)		<.001	

“Superusers,” defined as engaging with BeMe at least 2 SDs above the mean of the full sample (engaged >7 out of 30 days), were more likely to be female (351/557, 63% vs 7265/12,880, 56.4%), less likely to be male (38/557, 6.8% vs 1432/12,880, 11.12%; χ^2^_5_16.1; *P*=.007), and had lower PHQ-8 scores (15.04 vs 15.84; *F*_2,237_=5.08; *P*=.03) compared with nonsuperusers. There were no significant differences in age, 7-item Generalized Anxiety Disorder Questionnaire, WHO-5, or 4-item Perceived Stress Scale scores according to the BeMe superuser status. BeMe superusers were more likely to engage with live coaching (306/557, 54.9% vs 2237/12,880, 17.37%; χ^2^_5_=491.1, *P*<.001), clinical resources (267/557, 47.9% vs 2149/12,880, 16.68%; χ^2^_5_=353.6, *P*<.001), and crisis support resources (322/557, 57.8% vs 2180/12,880, 16.93%; χ^2^_5_=588.9, *P*<.001).

### Satisfaction and Helpfulness of Content and Features

Quick pulse surveys captured thumbs up or down ratings of different content and features of BeMe that were accessed by the participants. The surveys were launched at different times throughout the study period, resulting in varying sample sizes. Among the 8468 surveys, 5362 (63.32%) rated the BeMe activities as helpful for boosting mood. Among the 6072 surveys, 4408 (72.6%) rated the BeMe activities as helpful for coping with a big feeling. Completed by fewer participants owing to strategic placement of pulse surveys to prevent survey exhaustion and preserve the user experience, 91.66% (1044/1139) of adolescents planned to use a skill they learned from BeMe when coping with a stressor; 89.8% (158/176) rated the BeMe content as helpful; 86.2% (493/572) learned something about themselves from BeMe; 85.8% (235/274) felt relaxed after using BeMe; 84.3% (369/438) gained help with self-esteem; 83.4% (586/703) felt more hopeful; and 82% (46/56) felt less alone.

### Satisfaction and Helpfulness of Coaching

On average, the adolescents rated the BeMe coaching sessions 4.2 out of 5 stars (SD 1.2; n=893). Over four-fifths of the responses indicated that the sessions were helpful (747/894, 83.6%) and provided content that an adolescent would use (747/894, 83.6%; Table 4).

**Table 4:** Satisfaction and impact of digital activities and coaching.

Feature type				Values, N		Yes, n (%)
**Content**						
	Helpfulness		176		158 (89.77)	
	Self-efficacy		1139		1044 (91.66)	
	Hope		703		586 (83.36)	
	Self-identity		572		493 (86.19)	
	Self-esteem		438		369 (84.25)	
	Social connection		56		46 (82.14)	
	Relaxation		274		235 (85.77)	
**Interactive activities**						
	Helpfulness		8468		5362 (63.32)	
	Useful in coping with a big feeling		6072		4408 (72.6)	
**Coaching**						
	Helpfulness		894		747 (83.56)	
	Self-efficacy		894		747 (83.56)	
	**Overall rating**					
		5 stars	893		532 (59.57)	
		4 stars	893		163 (18.25)	
		3 stars	893		84 (9.41)	
		2 stars	893		34 (3.81)	
		1 star	893		78 (8.73)	

### Predictors of Satisfaction and Helpfulness

**Figure 2:**
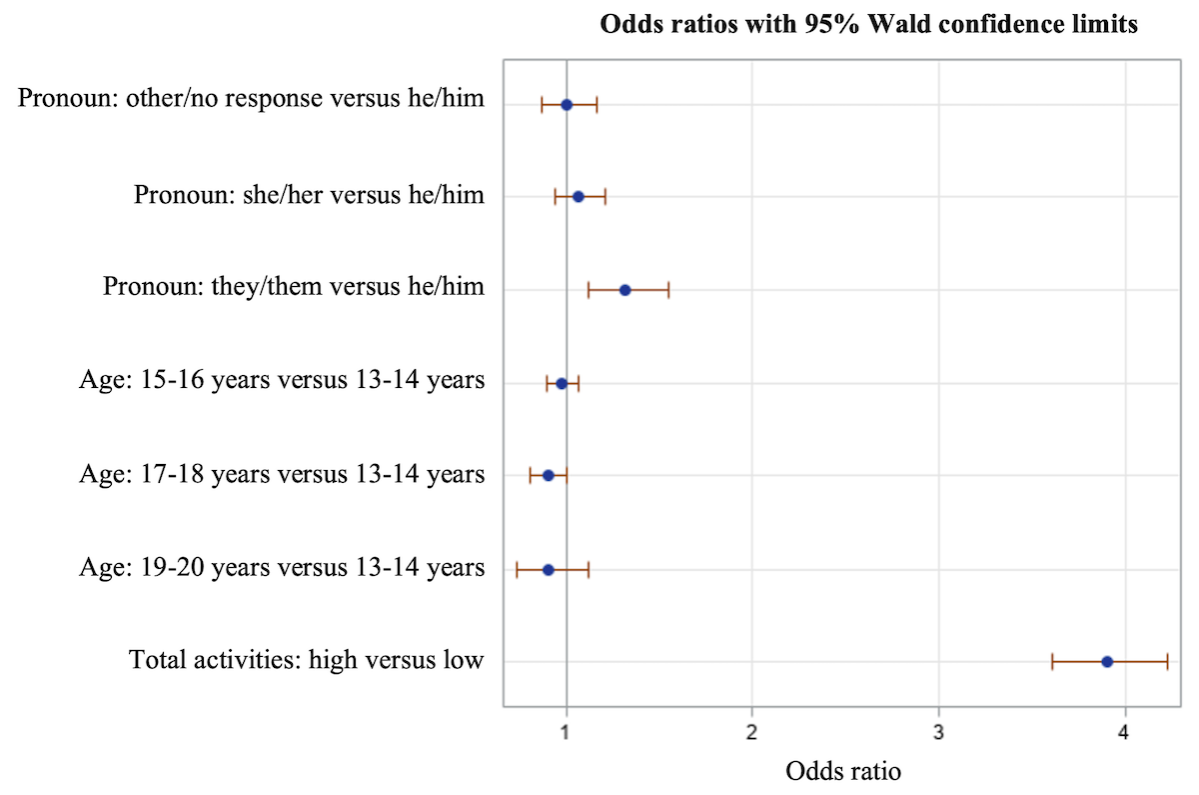
Odds ratios and CIs for a logistic regression model testing the effects of pronouns, age, and app engagement (high vs low activities over 30 days) on satisfaction (positive endorsement of at least 1 survey after a piece of content or interactive activity).

Logistic regression models were run to examine the predictors of BeMe satisfaction and the perceived helpfulness of content and interactive activities. Modeled outcomes were yes versus no or no response on surveys of satisfaction and perceived helpfulness. An initial model with inclusion of WHO-5 Well-being Index scores was nonsignificant, and we show a second model without these scores (Table 5). The tested predictor variables were gender identity in 4 categories (she/her as the reference group), age in 4 categories (13-14 years as the reference group), and high versus low total activities (<7 vs >7 activities). The only predictor that was significant was total activities, with those completing ≥7 activities being almost 3.9 times as likely to have indicated that they were satisfied with and found help from BeMe content or an activity compared with those with low total activities (*P*<.001; Figure 2; Table 5).

**Table 5:** Logistic regression model predicting at least 1 positive indicator of satisfaction or impact (N=13,421).

Effect			Odds ratio (95% CI)		*P* value
**Pronoun**					
	Other/no response vs he/him	1.004 (0.87-1.159)		.96	
	She/her vs he/him	1.066 (0.938-1.212)		.33	
	They/them vs he/him	1.315 (1.116-1.549)		.001	
**Age (years)**					
	15-16 vs 13-14	0.977 (0.896-1.065)		.59	
	17-18 vs 13-14	0.900 (0.809-1.002)		.06	
	19-20 vs 13-14	0.905 (0.730-1.120)		.36	
Total activities: high vs low			3.903 (3.607-4.224)		<.001

## Discussion

### Principal Findings

In the first large-scale evaluation of the acceptability and utility of the BeMe app, designed by and for adolescents, >6 times as many adolescents were enrolled than initially sought for the study. In 8 weeks, >13,000 adolescents accessed the app without the enticement of any financial incentive for using the app or completing within-app surveys. The adolescents reported interest in mood boosting, stress management, help with relationships, and discovering their identity, and most participants (11,005/13,421, 82%) identified multiple areas of interest. The great response suggests the demand for multifocal and multifunctional digital mental health programs among adolescents.

Characteristic of other mental health and digital program evaluations, BeMe attracted more adolescents who identified as female than male and notably attracted a sizable proportion of adolescents identifying as gender neutral (1391/13,421, 10.36%) or another gender (671/13,421, 5%). According to the literature, adolescent girls tend to be more willing to seek mental health treatment than boys [50] and are more likely to be attracted to wellness and mental health apps [51]. BeMe also attracted and engaged younger adolescents. Early adolescence is a major transitional period physically (puberty), environmentally (eg, transition to high school), and socially, when many new stressors arise [52] and when support may be particularly welcomed.

Across a 30-day evaluation of the BeMe app, participants’ use averaged >2 days, in over 8 sessions, engaging in >11 activities, with a great deal of variation, indicating diversity in adolescents’ needs and preferences. Notably, there was no in-app guidance toward a specific pathway; therefore, adolescents were able to gravitate toward the features that were most relevant to them. This was a conscious decision among the app’s designers and the BeMe Teen Advisory Board who worked together to create an experience that would focus on adolescent choice rather than explicit guidance. A better understanding of adolescent-guided pathways will allow for enhanced personalization over time and the ability to guide adolescents toward journeys that support their presentation (eg, clinical functioning) and their needs and preferences.

The observed level of engagement is satisfactory for a beta version of an app that does not ask or require participants to move in any particular pathway or journey through the app experience. For example, prior work has similarly found that users engage with certain app features just a couple of times, with 1 study observing that only 15.6% of unique app resources were engaged with at least once [53,54]. Another prior study of engagement with a mental health app that did not involve user prompts found that participants engaged in a total of 6 sessions on average, similar to the total of 7.94 sessions observed in this study [55].

Although the BeMe platform was initially designed to encourage exploratory self-navigation by teens (rather than a forced pathway), its developers were cautious about incorporating features in the initial design that could encourage excessive daily use, thereby avoiding overuse of the intervention. Social media platforms that also include content and live human connections (eg, messaging) have saturated the adolescent market and are being used in a way that prevents interactions with other areas of life for some teens (eg, school, in-person friend connections, and family relationships). This has likely contributed to a time-sink problem, whereby some teens report they are on social media “almost constantly” (35% in a 2022 Pew survey [16]) and that it would be difficult to give up social media (36% in the same survey). In contrast, the beta version of the BeMe app avoided some of the common features of social media platforms that promote constant use, such as algorithms designed to promote long-term content engagement [56] and social features that encourage social feedback and almost constant connections. There was an expectation that adolescents would use BeMe at variable rates, which was indeed found in this study. The findings of this study indicate that encouraging further engagement (eg, guidance toward assessment completion, personalized content pathways, and purposeful promotion of coaching sessions) could drive even further toward meaningful engagement.

A preliminary analysis of BeMe’s “superusers” confirmed active use among female adolescents and engagement in the live coaching, clinical service linkage, and crisis support features among those who used BeMe for >7 days in a month. A more detailed analysis of these users could help identify features they returned to use within the app to inform further app development, recruitment, onboarding, and impact, in line with previous work with adults [57]. A future examination of app use as it relates to changes in clinical symptoms and cognitive mediators of symptom incidence and course (eg, hope and self-esteem) will further inform targeted app development. Superusers also showed slightly lower PHQ-8 scores than those who used the app less often. Given that assessments could be completed at any time in a user’s journey, this could indicate either a positive impact of BeMe use on depression symptoms among superusers or less depression among superusers at the times they started to use the app. A more targeted journey for adolescents with depression will ensure that the experience supports them.

App engagement is greater among younger than older adolescents and among female and gender-neutral adolescents than male adolescents. BeMe’s constellation of services and support (eg, coaching) may be of greater interest to female adolescents and those who seek support for managing mood in particular. More work is needed to understand how best to reach and support male adolescents. Engagement patterns also indicate that there is potential for expanding app design, content, activities, and coaching features that resonate with older adolescents. BeMe could benefit from features that support developmental milestones of the older adolescent years (eg, graduating from high school, transitioning toward increasing independence in living situation, social interactions, work, and college).

Anxiety was the most common topic raised in coaching sessions, followed by relationships of multiple types, school, self-esteem, and depression. Developmental milestones of adolescence have, in many ways, been interrupted for this pandemic-stalled generation. Coaching may support adolescents to get back on track. The preponderance of sessions coded with the “other” topic suggests that the initial topic list should be expanded. Post hoc examination of sessions coded as other suggests that additional topics could include exploring adolescent identity or selfhood, attention or motivation, and social or conversational skills. Notably, the adolescents who engaged in the coaching sessions rated them highly, on average, 4.2 out of 5 stars. High ratings contribute to the promise of the paraprofessional model in delivering coping skill support digitally to adolescents and young adults [22].

Although a minority of the sample (1 in 10 or fewer) completed the well-being, anxiety, and depression assessments, a majority of those who self-selected to complete these measures indicated clinically concerning levels of distress, which supports the multiple offerings of BeMe, including individual counseling, safety planning, and clinical resources. BeMe can also be useful as a platform for tracking changes in anxiety and depressive symptoms over time using clinically validated measures with adolescents. Mood data from digital platforms such as BeMe can help to add nuance to the understanding of adolescent emotional experiences. Mood rating data from BeMe’s interactive mood rating feature indicate that most moods expressed while using the platform are lower arousal moods (eg, low, chill, and bored), and least endorsed moods are higher arousal moods (eg, excited and furious). This is consistent with prior literature showing that adolescents experience low-intensity emotions (both positive and negative) more frequently than high-intensity emotions, regardless of age [58]. A future investigation could develop a more nuanced understanding of the relationships among mood rating completion, adolescent individual characteristics (eg, age and gender), and clinical functioning (eg, depression and anxiety).

The single-item response measures throughout the intervention platform provide a unique way for adolescents to share feedback and provide a pulse on satisfaction and helpfulness. The responses were very positive for cognitive precursors aimed at reducing depression and anxiety such as hopefulness, perceived helpfulness, and the ability to cope with a big feeling. The likelihood of implementing these skills is high and warrants further study.

About one-fifth of the adolescents on the BeMe platform accessed each of the live supports, including coaching sessions, clinical resources, and crisis support tools. Digital support through a platform such as BeMe may be particularly appealing to a generation that exhibits decreased stigma regarding mental health challenges, while also displaying a growing mistrust of conventional mental health assistance in comparison with previous generations [59]. Similar rates of connection to coaching and crisis support as to traditional mental health services suggest that a platform like BeMe may be able to address challenges some adolescents have with accessing traditional clinical interventions (eg, need for caregiver consent and stigma) and foster greater trust and satisfaction with accessible alternatives. Crisis support use and high completion (1129/2502, 45.12%) of digital safety plans among those who access crisis resources highlight the utility of these features for a generation that is struggling with high and increasing suicidality [60], emergency department visits for suicidal behavior [61], and suicide completions [62]. Future investigations should examine the pathways between access to clinical and crisis support services through a digital platform such as BeMe, linkage to such services, and subsequent clinical functioning.

The multiple modalities that make up BeMe’s platform were designed to support across the acuity spectrum and offer options for engagement in multiple live features that are adolescent led (eg, coaching and crisis support). The beta version allowed unlimited engagement with 24/7 live crisis support and live coaching for 14 hours per day, but this pattern might need adaption as adolescents’ use of the live service and its impact on clinical functioning are assessed over time. The dissemination of a multimodal platform such as BeMe is best supported by organizations that support health at the population level (eg, health plans) or invest in the well-being of teens and their families (eg, employers of teens’ parents). BeMe’s Teen Advisory Board and other adolescents can inform ways to best iterate upon the delivery of live features (eg, coaching) for operational efficiency and dissemination at scale.

### Limitations

A study limitation is the incomplete data on measures of interest, given the self-driven nature of the BeMe app. Assessment completion was opportunistic, in that adolescents had to find the mood and well-being assessments or access content to trigger a pulse survey. The variety of information collected on participant characteristics was also limited to minimize burden in this initial evaluation of app use.

### Conclusions

Overall, BeMe is a promising example of the combination of digital and live interactive support in practice. This study contributes to the growing body of work demonstrating the utility and impact of the combination of digital and live human support in digital interventions [21,63], and demonstrated acceptability and utility in a large sample of adolescents. The positive responses to BeMe’s content, activities, and coaching service are encouraging. The next stage of evaluation will measure the changes in clinical functioning and well-being associated with BeMe use over time.

## Appendix

Multimedia Appendix 1 BeMe terms of service.

Multimedia Appendix 2 BeMe samples.

## Abbreviations

IV: independent variable
LGBTQIA: lesbian, gay, bisexual, transgender, queer, intersex, asexual, and similar minority
OR: odds ratio
PHQ-8: 8-item Patient Health Questionnaire
WHO-5: World Health Organization–Five Well-being Index

## Data Availability

The data sets generated or analyzed during this study are available from the corresponding author upon reasonable request.
