# Supplementary material for: Acceptability and Utility of a Smartphone App to Support Adolescent Mental Health (BeMe): Program Evaluation Study
Source: JMIR Mhealth Uhealth. 2023 Aug 28;11:e47183. doi: 10.2196/47183 (PMC10495844; doi:10.2196/47183)
Supplement: Multimedia Appendix 1 [file mhealth_v11i1e47183_app1.pdf]

BeMe Terms of Service aka ”The Legal Stuff.”

If you are or become at least 18 years or age, or if you are an emancipated minor, here is more “legal stuff” for you to read. This is an actual agreement that binds you, so please read it carefully.

AGREEMENT, ACKNOWLEDGEMENT, AND ACCEPTANCE OF TERMS
These Terms of Use ("Terms") govern your access to and use of our services, interfaces, and properties, which include but are not limited to websites, mobile applications, software, email, social media, and any other service or product contained or offered therein (collectively the "Service") that are owned or controlled by BeMe Health ("BeMe," "we," "us," and "our").

These Terms affect your legal rights, responsibilities, and obligations; govern your use of the Service; are legally binding; limit BeMe’s liability to you; and require you to indemnify BeMe and to settle certain disputes through arbitration. Your acceptance of, and compliance with, these Terms is a condition to your use of our Service. You understand and accept that we may, in our sole discretion, terminate your access to all or part of the Service at any time, with or without cause, and with or without notice.

We reserve the right to change these Terms at any time and at our sole discretion. Any changes to the Terms will be effective immediately upon postin, and you agree to the new posted Terms by continuing your use of the Service. It is your responsibility to check periodically for any changes we may make to these Terms. In addition, these Terms are written in the English language. We do not guarantee the accuracy of any translated versions of these Terms. To the extent any translated versions of these Terms conflict with the English language version, the English language version of these Terms will control.

By clicking "accept" or otherwise using the Service, you acknowledge that you have read, understand, and accept all terms and conditions contained within these Terms and our Privacy Policy. You agree that information provided by you in connection with the Service is governed by our Privacy Policy. If you don’t agree to these Terms or the Privacy Policy, don’t use the Service.

BeMe Health DOES NOT PROVIDE MEDICAL ADVICE, MEDICAL SERVICES, OR TREATMENT. The content of the Service, including without limitation, text, copy, audio, video, photographs, illustrations, graphics and other visuals, is for informational

purposes only and does not constitute professional medical advice, diagnosis, treatment, or recommendations of any kind by BeMe. You should always seek the advice of qualified health care professionals with any questions or concerns you may have regarding your individual needs and any medical or mental health conditions, and before stopping, starting, or modifying any treatment or modification.

THE SERVICE IS NOT FOR MEDICAL EMERGENCIES OR URGENT SITUATIONS. IF YOU ARE EXPERIENCING A MEDICAL EMERGENCY, YOU SHOULD DIAL "911" IMMEDIATELY. If you believe you have an emergency, call 9-1-1 immediately. BeMe is not a crisis support service -- if you need immediate support you can reach out to our 24/7 Crisis Hotline at 1-866-439-2363. You can also text Crisis Text Line by texting HOME to 741-741. Do not delay seeking medical advice or treatment based on anything that appears or does not appear in the Service.

ACCESS, SECURITY, AND RESTRICTIONS. You are prohibited from violating or attempting to violate the security of the Service, including, without limitation, by accessing data not intended for you or logging onto a server or an account which you are not authorized to access; attempting to probe, scan, or test the vulnerability of our system or network or to breach security or authentication measures without proper authorization; or using any scraper, crawler, spider, robot, or other automated means of any kind to access or copy data from the Service; or otherwise circumvent other measures we may use to prevent or restrict access to the Service. You agree not to use any device, software, or routine to interfere or attempt to interfere with the Service or any activity being conducted on the Service.

Violations of system or network security may result in civil or criminal liability. BeMe will investigate occurrences that may involve such violations and may involve, and cooperate with, law enforcement authorities in prosecuting users who are involved in such violations.

ELECTRONIC COMMUNICATIONS. When you use the Service, or send e-mails, messages, and other communications from your computer or mobile device to us, you are communicating with us electronically. You consent to receive communications from us electronically. You agree that (a) all agreements and consents can be signed electronically and (b) all notices, disclosures, and other communications that we provide to you electronically satisfy any legal requirement that such notices and other communications be in writing.

CONSENT TO RECEIVE CALLS AND TEXT MESSAGES. By providing your mobile number to us, you agree to be contacted by or on behalf of BeMe at the mobile number

you have provided, including via phone call or text message, to receive transactional communications relating to the Service. You recognize and acknowledge that text messaging is inherently less secure of a method of communication and agree to receive text messages regardless of the level of security associated with them. For help regarding the communications we exchange with you using your phone number, text the word HELP to [insert contact number].

OWNERSHIP OF SERVICE. The Service and all of its content, including without limitation all copyrights, patents, trademarks, service marks, and trade names, as well as all logos, text, design, graphics, logos, icons, images, audio clips, downloads, interfaces, code and software, as well as the selection and arrangement thereof, and all other intellectual property (collectively referred to as the "Content"), are all proprietary and owned or controlled by BeMe, our licensors, and certain other third parties.

All right, title, and interest in and to the Content available through the Service is the exclusive property of and owned by BeMe, our licensors or certain other third parties, and is protected by United States and international copyright, trademark, trade dress, patent or other intellectual property and unfair competition rights and laws to the fullest extent possible.

All rights not expressly granted to you in these Terms are reserved and retained by BeMe and its licensors, suppliers, publishers, rights holders, and other content providers. Accordingly, you may not modify, copy, distribute, reproduce, publish, retransmit, disseminate, rent, lease, loan, sell, publish, broadcast, display, circulate, or use the Content, in whole or in part, for any purpose that has not been authorized or approved in writing by BeMe, including but not limited to commercial purposes. You are also strictly prohibited from publicly displaying the Content; attempting to decompile or reverse engineer the Content; removing any copyright, trademark, or other proprietary notations from the Content; framing or utilizing framing techniques to enclose or deep-link to the Content; applying metatags or "hidden text" to the Content; or otherwise infringing upon the intellectual property rights of BeMe or misusing the Site or Service.

BeMe owns and uses several trademarks on the Service, including but not limited to: [insert your trademark/service mark here].

Nothing contained in the Service should be construed as granting, by implication, estoppel, waiver or otherwise, any license or right to use any intellectual property, including without limitation any trademarks, service marks or logos displayed on the Site, without the express written consent of BeMe or the third-party owner of such intellectual property. The Service may contain other proprietary notices and copyright

information, the terms of which you agree to follow by using the Service. Additionally, you acknowledge and accept that BeMe may in its sole discretion delete any information provided by you that it deems to be fraudulent, abusive, defamatory, obscene, or in violation of any intellectual property or ownership rights of any other person or entity.

Some features of the Service may now or in the future allow you to provide content, such as written comments or reviews, to be published or displayed on public areas of the Service ("User Content"). All content submitted by you to the Service may be retained by us indefinitely, even after you terminate your account. By submitting any User Content, you grant to BeMe a perpetual, worldwide, irrevocable, non-exclusive, royalty-free license to use the User Content in any manner, including to create derivative works, without any compensation or notice to you. Your User Content may be posted and transmitted to others at your own risk. We cannot control the actions of other Users of the Site with whom you may choose to share your User Content.

ACCURACY OF INFORMATION; FUNCTIONALITY. BeMe makes no representations, warranties, or guarantees whatsoever as to the correctness or accuracy of content in the Service. It is possible that the Service could include typographical errors, inaccuracies, or other errors, and that unauthorized additions, deletions, and alterations could be made by third parties. In the event that an inaccuracy arises, please inform BeMe so that it can be corrected.

BeMe shall have no responsibility or liability for information or content posted to the Service from any non-BeMe- affiliated third party.

BeMe reserves complete and sole discretion with respect to the operation of the Service. We may withdraw, suspend, or discontinue any functionality or feature of the Service. We are not responsible for transmission errors, corruption, or compromise of information carried over local or interchange telecommunications carriers. We are not responsible for maintaining information arising from use of the Service. We reserve the right to maintain, delete, or destroy all communications or information posted or uploaded to the Service in accordance with our internal record retention or destruction policies.

LINKS TO OTHER MATERIALS. The Service may provide links to other third-party websites ("Linked Sites"). BeMe has not reviewed information on the Linked Sites, does not maintain any of the Linked Sites, and cannot control the completeness, accuracy, or security of the content contained on any Linked Site. The content of, including materials

and information contained on, any Linked Site is solely the responsibility of the provider of that Linked Site.

The views and opinions expressed in Linked Sites are those of the authors or third parties and do not necessarily reflect the official policy or position of BeMe. The inclusion of any link to a Linked Site, and any references to any names, marks, products, or services of any third parties, third-party information, or Linked Sites are provided solely as a convenience to you, and do not constitute or imply an endorsement, sponsorship or recommendation of, or affiliation with, the third party or its products and services.

If you decide to access any Linked Site, you do this entirely at your own risk. BeMe makes no representation or warranty as to any Linked Site, content, products or services, and you agree that BeMe shall not be responsible or liable, directly or indirectly, for any damage or loss caused or alleged to be caused by or in connection with use of or reliance on any such third-party content, products, or services available on or through any Linked Site or similar resource.

AS PERMITTED BY APPLICABLE LAW, WE SHALL UNDER NO CIRCUMSTANCES BE LIABLE FOR ANY DIRECT, INDIRECT, INCIDENTAL OR SPECIAL LOSS, OR OTHER DAMAGE, WHETHER ARISING FROM NEGLIGENCE, BREACH OF CONTRACT, DEFAMATION, INFRINGEMENT OF COPYRIGHT, OR OTHER INTELLECTUAL PROPERTY RIGHTS, CAUSED BY THE EXHIBITION, DISTRIBUTION, OR EXPLOITATION OF ANY INFORMATION OR CONTENT CONTAINED WITHIN THESE THIRD-PARTY SERVICES.

USER INFORMATION. If you submit, upload, post or transmit any health information, medical history, conditions, problems, symptoms, requests, comments, ideas, suggestions, information, files, videos, images, or other materials or personal information to the Service ("User Information"), you agree not to provide any User Information that (1) is false, inaccurate, defamatory, abusive, libelous, unlawful, obscene, threatening, harassing, fraudulent, pornographic, or harmful, or that could encourage criminal or unethical behavior; (2) violates or infringes the privacy, copyright, trademark, trade dress, trade secrets or intellectual property rights of any person or entity; or (3) contains or transmits a virus, Trojan horse, time bomb, malware, or other computer programing or code that is designed or intended to damage, destroy, intercept, download, interfere, manipulate, or otherwise interrupt or expropriate the Service, personal information, software, equipment, servers, or facilitate or promote hacking or similar conduct. You represent and warrant to BeMe that you have the legal right and authorization to provide all User Information to the Service.

USER RESTRICTIONS. You agree not to:

1. contact other users of the Service through unsolicited e-mail, telephone calls, mailings, or any other method of communication;
2. Impersonate or misrepresent your identity or falsely state or misrepresent your affiliation with a person or entity;
3. Tamper, hack, spoof, copy, modify, or otherwise corrupt the administration, security, or proper function of the Service;
4. Use robots or scripts, or attempt to reverse engine, reverse assemble, reverse compile, decompile, disassemble, translate, or otherwise alter, defraud, or create false results from any executable code;
5. Have any antivirus or antispyware software running that is set to override the internet browser's cookies setting;
6. Incorrectly identify the sender of any message transmitted to the Service by altering the attribution or origin of electronic mail, messages, or posting;
7. Harvest or collect personal health information about any other individual who uses the Service;
8. Otherwise engage in or promote any act or activity contrary to any applicable local, state, or federal law.

YOU AGREE TO DEFEND, INDEMNIFY, AND HOLD HARMLESS BEME FROM AND AGAINST ALL THIRD-PARTY CLAIMS, DAMAGES AND EXPENSES, INCLUDING REASONABLE ATTORNEYS' FEES, AGAINST OR INCURRED BY US ARISING OUT OF ANY USER INFORMATION YOU UPLOAD TO OR TRANSMIT THROUGH THE SERVICE OR YOUR VIOLATION OF THE USER RESTRICTIONS.

COPYRIGHT POLICY. BeMe complies with the copyright notice-and-takedown procedures set out in the United States Digital Millennium Copyright Act (DMCA), which applies to content reported and removed for violating U.S. copyrights. Please note that any notice or counter-notice you submit must be truthful and must be submitted under penalty of perjury. A false notice or counter-notice may give rise to personal liability. You may therefore want to seek the advice of legal counsel before submitting a notice or a counter-notice. We may share any notices and counter-notices submitted to us with others including your contact information, and by submitting any notices, you agree you have no expectation of privacy in your submission.

For more information on submitting a notice or counter-notice, contact us at:

Copyright Designated Agent

Copyright Agent
c/o BeMe Health, Inc
295 Ocean Boulevard
Marblehead, MA 01945
Email: copyright@bemehealth.com

DISCLAIMER AND LIMITATION OF LIABILITY. ALL SERVICES, INFORMATION, GOODS, AND MATERIALS MADE AVAILABLE THROUGH THE SERVICE ARE PROVIDED TO YOU "AS IS" WITHOUT ANY EXPRESS REPRESENTATIONS OR WARRANTIES OF ANY KIND, AND WE DISCLAIM ALL STATUTORY OR IMPLIED REPRESENTATIONS, WARRANTIES, TERMS, AND CONDITIONS WITH RESPECT TO ALL SERVICES, INFORMATION, GOODS, AND MATERIALS MADE AVAILABLE THROUGH THE SERVICE, INCLUDING THE REPRESENTATIONS AND WARRANTIES OF SATISFACTORY QUALITY, MERCHANTABILITY, FITNESS FOR A PARTICULAR PURPOSE, NONINFRINGEMENT, AND TITLE. WE MAKE NO REPRESENTATION OR WARRANTY THAT THE SERVICE (OR ANY PART THEREOF) WILL BE ACCURATE, COMPLETE, OR ERROR-FREE. WE MAKE NO REPRESENTATION OR WARRANTY THAT ANY PARTICULAR SOFTWARE OR HARDWARE WILL BE COMPATIBLE WITH THE SERVICE, AND YOU HEREBY AGREE THAT IT IS YOUR SOLE RESPONSIBILITY TO (A) OBTAIN AND PAY FOR ANY SOFTWARE, HARDWARE AND SERVICES (INCLUDING INTERNET CONNECTIVITY) NEEDED TO ACCESS AND USE THE SITE AND (B) ENSURE THAT ANY SOFTWARE, HARDWARE, AND SERVICES THAT YOU USE WILL FUNCTION CORRECTLY WITH THE SERVICE. YOU AGREE THAT YOU MUST EVALUATE, AND THAT YOU BEAR ALL RISKS ASSOCIATED WITH, THE USE OF THE SERVICE, INCLUDING ANY RELIANCE ON THE ACCURACY, COMPLETENESS, OR USEFULNESS OF ANY INFORMATION OR MATERIALS MADE AVAILABLE THROUGH THE SERVICE.

WE WILL NOT BE LIABLE FOR ANY INDIRECT, INCIDENTAL, CONSEQUENTIAL, SPECIAL, EXEMPLARY, OR PUNITIVE DAMAGES OF ANY KIND IN CONNECTION WITH THE SERVICE, NOR FOR ANY DAMAGES FOR LOSS OF PROFITS, LOSS OF USE, LOSS OF DATA, LOSS OF OTHER INTANGIBLES, LOSS OF SECURITY OF INFORMATION YOU HAVE PROVIDED IN CONNECTION WITH YOUR USE OF THE SERVICE, OR UNAUTHORIZED INTERCEPTION OF ANY SUCH INFORMATION BY THIRD PARTIES, EVEN IF ADVISED IN ADVANCE OF SUCH DAMAGES OR LOSSES. FURTHER, WE WILL NOT BE LIABLE FOR DAMAGES OF ANY KIND RESULTING FROM YOUR USE OF THE SERVICE OR FROM ANY INFORMATION OR MATERIALS ON THE SERVICE.

YOUR SOLE AND EXCLUSIVE REMEDY FOR DISSATISFACTION WITH THE SERVICE IS TO STOP USING THE SERVICE. OUR MAXIMUM LIABILITY FOR ALL DAMAGES, LOSSES, AND CAUSES OF ACTION, WHETHER IN CONTRACT, TORT (INCLUDING, WITHOUT LIMITATION, NEGLIGENCE), OR OTHERWISE SHALL BE THE TOTAL AMOUNT, IF ANY, PAID BY YOU TO US TO ACCESS AND USE THE SERVICE.

IT IS POSSIBLE THAT APPLICABLE LAW MAY NOT ALLOW FOR LIMITATIONS ON CERTAIN IMPLIED WARRANTIES OR EXCLUSIONS OR LIMITATIONS OF CERTAIN DAMAGES; SOLELY TO THE EXTENT THAT SUCH LAW APPLIES TO YOU, SOME OR ALL OF THE ABOVE DISCLAIMERS, EXCLUSIONS, OR LIMITATIONS MAY NOT APPLY TO YOU, AND YOU MAY HAVE ADDITIONAL RIGHTS. IF APPLICABLE LAW PROHIBITS THE LIMITATION OR EXCLUSION OF A PARTY'S LIABILITY WITH RESPECT TO DEATH OR PERSONAL INJURY CAUSED BY SUCH PARTY'S NEGLIGENCE, FRAUD, OR ANY OTHER MATTER, THEN SUCH PARTY'S LIABILITY WILL NOT BE LIMITED OR EXCLUDED TO THE EXTENT OF SUCH PROHIBITION UNDER SUCH APPLICABLE LAW.

INDEMNIFICATION. Except to the extent prohibited under applicable law, you agree to indemnify, defend, and hold harmless BeMe, its officers, directors, employees, and agents, and any affiliates from and against any and all claims, losses, liability, damages, costs, or expenses, including reasonable attorneys' fees and costs, that may arise from or in connection with (a) your use of, or activities in connection with, the Service or (b) any violations of these Terms by you. If you fail to promptly indemnify and defend a covered claim, BeMe shall have the right to defend itself, and in such case, you shall promptly reimburse BeMe for all of its associated costs and expenses.

GOVERNING LAW. By accessing the Service, you agree that the statutes and laws of the United States and the state of Florida, USA, without regard to conflicts of laws principles, will apply to all matters relating to use of the Service. You further agree that any litigation, action, or proceeding arising out of or related to these Terms shall be subject to the sole and exclusive jurisdiction of the state of Florida and that venue shall be in an appropriate state or federal court located in Miami-Dade County, Florida. You hereby submit to the jurisdiction and venue of said courts and consent to service of process by email in any legal proceeding.

CLASS ACTION WAIVER. YOU AND BEME AGREE THAT EACH PARTY MAY BRING CLAIMS AGAINST THE OTHER ONLY ON AN INDIVIDUAL BASIS AND NOT AS A PLAINTIFF OR CLASS MEMBER IN ANY PURPORTED CLASS OR REPRESENTATIVE ACTION OR PROCEEDING.

THIRD PARTY RIGHTS. Unless expressly stated in these Terms, nothing herein is intended to confer any rights or remedies on any persons other than you and BeMe. Moreover, nothing in these Terms is intended to relieve or discharge the obligations or liability of any third persons to you or BeMe, nor shall any provision give any third parties any right of subrogation or action against you or BeMe.

ASSIGNMENT. You may not assign, transfer, or delegate these Terms or any part thereof without BeMe's express written consent. BeMe may freely transfer, assign, or delegate all or any part of these Terms, and any rights or duties hereunder or thereunder. These Terms will be binding upon and inure to the benefit of the heirs, successors, and permitted assignees of the parties.

FORCE MAJEURE. We will not be deemed to be in breach of these Terms or liable for any breach of these Terms or our Privacy Policy due to any event or occurrence beyond our reasonable control, including without limitation acts of God, terrorism, war, invasion, failures of any public networks, electrical shortages, earthquakes or floods, civil disorder, strikes, fire, or other disaster.

MISCELLANEOUS TERMS. These Terms and other documents cited in this agreement constitute the entire agreement between you and BeMe and govern your use of the Service, superseding any prior agreements between you and BeMe relating to your use of the Services. If any provision of these Terms is held to be invalid by any law, rule, order, or regulation of any government or by the final determination of any state or federal court, such invalidity shall not affect the enforceability of any other provision of the Terms. The failure of BeMe to exercise or enforce any right or provision of the Terms shall not constitute a waiver of such right or provision.

CONTACT US. If you have any questions about these Terms, please write to or call us at: privacy@bemehealth.com. BeMe does not guarantee that it will receive such communications timely and accurately and shall not be legally obligated to read, act on, or respond to any such email or other information.

EFFECTIVE DATE: JUNE 12, 2022
