# Supplementary figures and images for: Acceptability and Utility of a Smartphone App to Support Adolescent Mental Health (BeMe): Program Evaluation Study
Source: JMIR Mhealth Uhealth. 2023 Aug 28;11:e47183. doi: 10.2196/47183 (PMC10495844; doi:10.2196/47183)

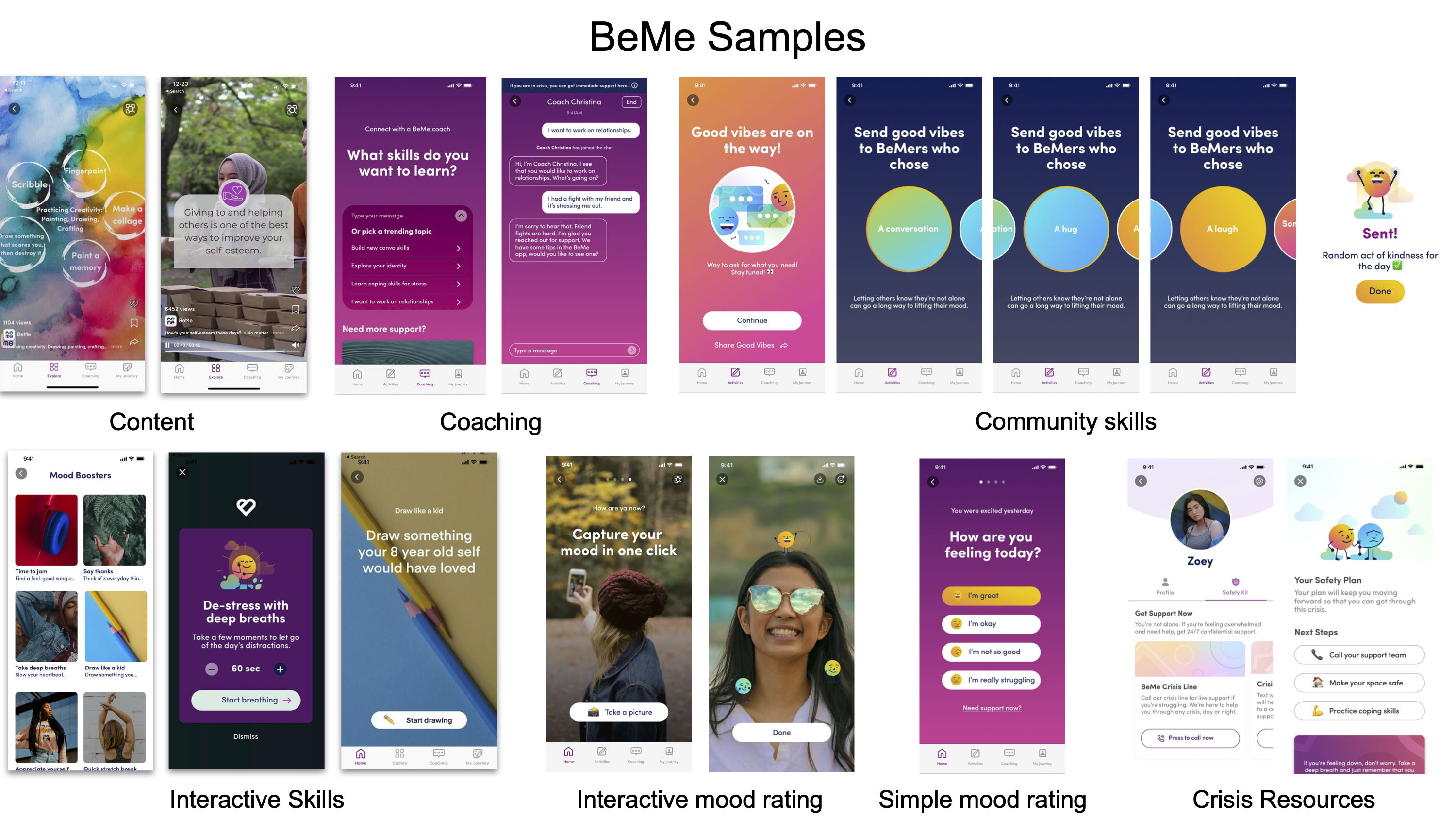

Supplement: Multimedia Appendix 2 [file mhealth_v11i1e47183_app2.png]
